# Materia Medica and Therapeutics

**Published:** 1893-02

**Authors:** 


MATERIA MEDICA AND THERAPEUTICS.—A Manual for Students and Practitioners; L. F. Warner, M. D., Attending Physician St. Bartholomew's Dispensary, New York. Philadelphia, Lea Brothers & Co. Price $1.00.

INTENDED more particularly for the use of students, the aim of the author, in the preparation of this little manual has been to omit all elaborate discussion, and to state tersely, yet fully, the essential points. In it is considered only such drugs as will be most frequently used by the practitioner, and those which have recently recommended themselves to the notice of therapeutists. C. E. J.

